# Work-related outcomes in individuals with and without lower limb osteoarthritis: an online survey

**DOI:** 10.1186/s12889-023-16723-3

**Published:** 2023-09-29

**Authors:** Yousef S. Alyousef, Venerina Johnston, Michelle D. Smith

**Affiliations:** 1 The University of Queensland, School of Health and Rehabilitation Sciences, Physiotherapy, Brisbane, QLD Australia; 2 College of Applied Medical Sciences, Majmaah University, Al Majmaah, Saudi Arabia; 3 School of Health and Medical Sciences, University of Southern Queensland, Ipswich, QLD Australia

**Keywords:** Osteoarthritis, Lower limb, Work, Absenteeism, Presenteeism

## Abstract

**Objective:**

While osteoarthritis (OA) affects people who are still participating in the workforce, there is limited data about the impact of OA on work. The aim of this study was to compare work participation in individuals with and without lower limb OA.

**Methods:**

This cross-sectional study included workers with (*n* = 124) and without (*n* = 106) lower limb OA. Work participation was assessed as work status (full/part time work), work ability (Work Ability Index (WAI)), absenteeism and presenteeism (World Health Organization’s Health and Work Performance Questionnaire (WHO-HPQ)), and perceived difficulties meeting work demands (Work Role Functioning Questionnaire (WRFQ)). The data were analyzed using an analysis of covariance with age, body mass index and physical job demands included as covariates.

**Results:**

Work ability was poorer (*p* < 0.001) and loss of work performance (*p* < 0.001) was higher among workers with OA than healthy controls. There was no statistical difference in absenteeism or overall ability to meet work demands between participants with and without lower limb OA. However, workers with lower limb OA had more difficulty with work scheduling demands (*p* = 0.05) and physical demands (*p* = 0.003) than healthy workers.

**Conclusion:**

Lower limb OA was associated with poorer work ability, loss of work performance and difficulty in meeting physical and work scheduling demands. Health professionals and employers should consider these challenges when managing individuals with lower limb OA and supporting them to remain in the workforce.

## Introduction

Osteoarthritis (OA) is a prevalent condition with significant implications to individuals and society [1]. It commonly effects the lower limb, particularly the hip and knee [2], and is associated with high pain and disability [2–4]. The Australian Institute of Health and Welfare estimates that OA accounts for 3% of the total burden of disease in Australia and 28% of disease expenditure on musculoskeletal conditions is on OA [5]. With recognition that OA commonly affects people of working age [6], it has been suggested that indirect costs related to lost work productivity is a significant contributor to the economic burden of OA [7].

There is limited evidence about the impact of lower limb OA on work. The majority of literature focuses on work status with less attention to other work-related outcomes, such as absenteeism and presenteeism [8]. Research is also limited to people with hip and knee OA. No research on work-related outcomes has been done on foot and ankle OA, which have similar pain and disability to hip and knee OA [4, 9]. A 2011 systematic review concluded that knee and hip OA had a mild negative impact on work participation [10] and a 2023 systematic review identified low absenteeism and high presenteeism in individuals with OA of any joint [8]. In addition, case–control studies report lower rates of employment in people with hip and knee OA compared to pain-free controls [11, 12]. There is suggestion in the literature that individuals with hip and knee OA have more absenteeism from work than those without OA [13, 14] and that many workers with OA leave the workforce prior to the usual retirement age [15, 16]. These suggestions are concerning as work is a significant part of a person’s life, providing financial and social benefits [17].

Globally, with an ageing population and an increase in the retirement age [18], the numbers of people affected by OA in the workforce is likely to increase [19]. At a time of national employment shortages in many countries [20–22], it is imperative to retain people at work to maintain labour force productivity and individual financial well-being. In order to do this, it is important to clarify the impact of OA on work and identify work-related issues that need to be addressed. This study aims to compare work-related outcomes (e.g., work ability, absenteeism, productivity loss and difficulty in meeting work demands) in people with and without lower limb OA.

## Method

### Design

This cross-sectional case–control online survey was carried out between May 2020 and February 2022.

### Participants

Working individuals with and without lower limb OA in Australia were recruited via social media (e.g., Facebook, Twitter), online newsletters, websites (e.g., Arthritis Australia) and electronic advertisements/posters placed around the university. Study advertisements invited individuals with and without lower limb OA who were 35 years of age or older and employed in paid work or self-employed to complete in an online survey about how their joint pain effects them at work. Individuals who responded to study advertisements were directed to an online survey to assess eligibility. Inclusion criteria for all participants were: aged ≥ 35 years, employed in paid work or self-employed, and able to read and write in English. Additional inclusion criteria for participants with lower limb OA were hip, knee, ankle or foot pain for at least three months and one of the following: self-report of a diagnosis of hip, knee, ankle or foot OA by a healthcare practitioner, or a clinical diagnosis of OA based on the National Institute of Health and Care Excellence (NICE) guidelines (i.e., ≥ 45 years of age, activity-related joint pain, and no or minimal (resolves within 30–60 min) morning joint-related stiffness) [23]. Study participants without lower limb OA were required to not experience any bodily pain. Study exclusion criteria were pregnancy; previous joint replacement surgery; receiving treatment for cancer; any neurological, vestibular, or systemic conditions; and pain in areas of the body that was worse than that at the affected joint (for the lower limb OA group).

### Data collection and measures

Data was collected using an online survey hosted on the Qualtrics_®_^XM^ platform (Provo, Utah, USA). The survey was developed following a review of the literature on work-related outcomes in people with lower limb OA [24] and piloted by members of the research team before distribution. Several work-related outcomes were collected as there is no agreed-upon gold standard for work participation, which is a multi-dimensional construct [25]. Three validated scales were included: the Work Ability Index (WAI), Health Organization’s Health and Work Performance Questionnaire (WHO-HPQ) and Work Role Functioning Questionnaire (WRFQ).

The WAI is a valid and reliable questionnaire used to assess work ability in relation to the physical and mental demands of a job [26]. The WAI includes seven items with individual items resulting in a cumulative score from 7–49. Work ability is categorised as: poor (7–27 points), moderate (28–36 points), good (37–43 points) and excellent (44–49 points). The WAI has been used in people with OA [27].

Self-reported absenteeism (days taken off work) and presenteeism (loss of work performance) were assessed using the 7-item WHO-HPQ [28]. The WHO-HPQ has good reliability and validity and is widely used as an outcome measure for working populations to quantify productivity loss [29]. Estimated hours lost over the last four weeks was assessed with four items and was used to calculate absolute absenteeism in hours per month. A higher score indicates a higher amount of absenteeism. Presenteeism was assessed with three questions as a measure of actual performance in relation to possible performance. Participants were asked to rate the performance of most workers in a similar job to theirs, their own performance over the past year or two, and their overall job performance on the days they worked during the past 4 weeks. Questions to calculate absolute presenteeism were answered on a numerical rating scale from 0–10 where 0 is ‘worst job performance’ and 10 is ‘top job performance’. A higher score indicates a lower amount of lost performance.

The WRFQ was used to measure the degree of difficulty in performing work demands due to physical health or emotional problems over the past four weeks. This questionnaire is applicable in the general working population, irrespective of type of work [30], and has validity and reliability in clinical and general working populations [31]. The WRFQ consists of 27 items across five domains—work scheduling (e.g. *‘Get going easily at the beginning of the workday*’), output (e.g. ‘*Work fast enough’)*, physical demands *(e.g. ‘Sit, stand, or stay in one position for longer than 15 min while working’)*, mental and social demands *(e.g. ‘Concentrate on your work’)*, and flexibility demands *(e.g. ‘Perform multiple tasks at the same time’)* [31]. For each item, the individual is asked to indicate the degree of difficulty they have performing their job demands on a scale from 0 (difficult all of the time) to 4 (no difficulty). Scores are averaged and multiplied by 25 for an overall score and a score for each domain out of 100, with higher scores indicating better work role functioning. Scores 0 – 90 indicate ‘working but only able to meet the demands of the job less than 90% of the time'; scores > 90 to ≤ 95 are considered to indicate ‘good work functioning’, while a score > 95–100 is indicative of ‘successful work functioning’ [32].

The survey collected the following demographic information to describe the study sample: age, sex, weight, height, level of education, occupation, physical effort at work, work hours, full/part-time work status, annual income category, number of comorbidities, physical activity level and severity of lower limb joint pain (for participants with OA). Participants were asked to nominate their occupation based on the Australian and New Zealand Standard Classification of Occupations [33]. This classification included eight main categories (e.g., manager, professional, technician/trades worker, community/personal service worker, clerical/administrative worker, sales worker, machinery operator/driver, and labourer) and an option for “other”. These eight categories were collapsed to three main classifications based on job demands: 1) manager/professional, including managers and professionals; 2) trade/manual workers, including technician/trades worker, machinery operator/driver, and labourer; and 3) service workers, including community/personal service worker, clerical/administrative worker and sales worker. Physical effort associated with work was evaluated using the Borg scale in which participants rated their perceived exertion during work on a 6–20 scale [34]. This scale has been validated against observer-rated physical effort in the workplace [35]. The number of comorbidities was calculated from the WAI, which asks participants to nominate if they have any of 13 physician-diagnosed disorders (e.g., cardiovascular disease). Participants rated the worst pain they experienced in their affected lower limb joint in the past week on an 10-point numerical rating scale (NRS) anchored with ‘no pain’ at 0 and ‘worst pain imaginable’ at 10 [36]. The International Physical Activity Questionnaire (IPAQ) short form (7 items) was used to evaluate physical activity level [37], which was categorised as ‘low’, ‘moderate’, or ‘high’.

### Statistical analysis

Data were analysed using the Statistical Package of Social Science (SPSS; Version 26, IBM Corporation, Armonk, NY). Data were tested for normality by inspection of histograms, quantile–quantile plots, and the Shapiro–Wilk test. While data were not normally distributed, comparison of non-parametric (Kruskal–Wallis test) and parametric (Analysis of Variance (ANOVA)) analyses indicated no difference in statistical findings between methods. Thus, to enable inclusion of covariates in analyses (age, body mass index (BMI) and physical job demands), an analysis of covariance (ANCOVA) was used to compare the WAI, WHO-HPQ and WRFQ between participants with and without lower limb OA. Descriptive statistics (independent t-test for continuous variables and Chi-square test for dichotomous variables) were used to describe the characteristics of the study participants. Continuous data are reported as median and interquartile range (first and third quartiles) with *P* value using Kruskal–Wallis test, and adjusted *P* value using ANCOVA. Statistical significance was set at *p* < 0.05.

## Results

### Study participants

**Fig. 1:**
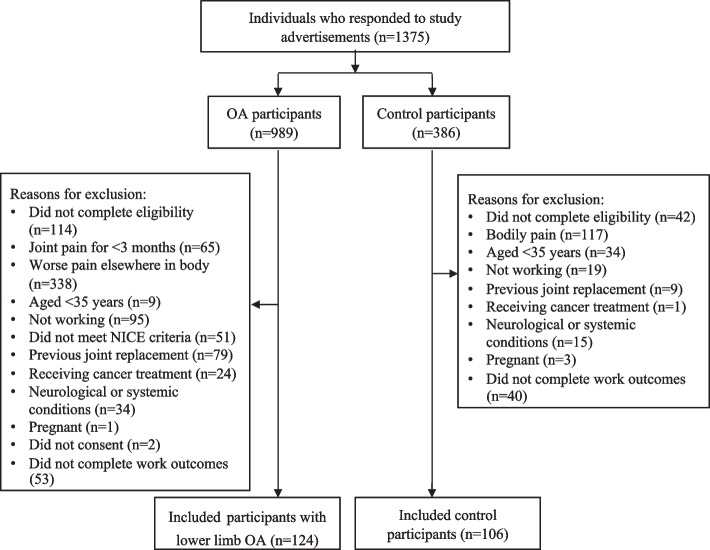
Flow chart outlining participant recruitment

A total of 1375 individuals responded to study advertisements. After excluding individuals who did not complete the eligibility assessment (*n* = 156) and those who did not meet the eligibility criteria (*n* = 896), 323 individuals were eligible to participate. Ninety-three individuals did not provide any data on work-related outcomes, leaving 124 individuals with lower limb OA and 106 control individuals who participated in the study (*n* = 230) (Fig. 1). In the lower limb OA group, 51 participants reported one joint affected, 32 reported two joints affected and 18 reported three or more joints affected (missing data for 23 participants). Sixty-two participants had knee OA, 19 had hip OA, 9 had ankle OA and 11 had foot OA. The mean (SD) worst pain intensity over the last week was 6.9 (2.1) out of 10.

Participants with and without lower limb OA were similar in age, BMI, education level, occupational category, work status (full or part-time work), annual income, and physical activity level (Table 1; all *p* ≥ 0.1). There was a greater proportion of females in the lower limb OA group (*p* = 0.01), and participants with OA had more comorbidities (*p* < 0.001) and were employed in jobs that required higher physical effort than controls (Table 1; *p* = 0.01). When work demands (e.g., mental, physical, and both) were compared between groups, healthy controls were more likely to hold jobs characterized by mental demand (OA: 44.3%, *n* = 55; controls: 60.4%, *n* = 64) while individuals with OA were more likely to hold jobs characterized by both physical and mental demands (OA: 48.4%, *n* = 60; controls: 28.3%, *n* = 30; *p* < 0.008).

**Table 1:** Characteristics of participants with lower limb osteoarthritis (OA) and controls

Characteristic	OA group (*n* = 124)	Control group (*n* = 106)	*P* value
**Age**, years (Mean **(**SD))	57.6 (9.0)	55.3 (9.5)	0.8^a^
**Sex**, # female	99 (79.8)	69 (65.1)	0.01^b^
**BMI,** kg/m2 (Mean **(**SD))	29.8 (7.7)	28.7 (9.7)	0.3^a^
**Education level**			
University degree or higher	71 (57.3)	62 (58.5)	0.8^b^
Diploma	23 (18.5)	20 (18.8)	
Certificate I-IV	16 (12.9)	11 (10.4)	
Primary/secondary education	14 (11.3)	13 (12.3)	
**Occupational category**			
Manager/professional	64 (51.6)	63 (59.4)	0.6^b^
Trade/manual workers	20 (16.1)	22 (20.8)	
Service workers	40 (32.3)	21 (19.8)	
**Physical effort**, 6–20 (Mean (SD))	10.8 (3.2)	10.1 (2.8)	0.01^b^
**Work status**			
Full time ≥ 35 h per week	70 (56.5)	70 (66)	0.1^b^
Part time < 35 h per week	54 (43.5)	36 (34)	
Total hours per week (Mean (range))	34.3 (3–75)	36.8 (2–90)	0.3^a^
Annual income*			
Prefer not to answer	28 (22.6)	14 (13.2)	0.4^b^
$1-$31,199	22 (17.7)	15 (14.2)	
$31,200-$51,999	13 (10.5)	19 (17.9)	
$52,000-$77,999	17 (13.7)	21 (19.8)	
$78,000-$103,999	24 (19.4)	15 (14.2)	
≥ $104,000	20 (16.1)	22 (20.7)	
**Number of comorbidities**			< 0.001^b^
0	7 (5.7)	61 (57.5)	
1	22 (17.7)	24 (22.6)	
2	29 (23.4)	13 (12.3)	
3	29 (23.4)	4 (3.8)	
≥ 4	37 (29.8)	4 (3.8)	
**Physical activity** (IPAQ)			0.5^b^
Low level	17 (18.9)	13 (14.0)	
Moderate level	29 (32.2)	36 (38.7)	
High level	44 (48.9)	44 (47.3)	

All results are presented as number (%) unless otherwise stated

^a^Independent-sample t-tests

^b^Chi-square tests

*SD* Standard deviation, *Income in AUD

### Work-related outcomes

Individuals with lower limb OA have lower overall scores on the WAI than healthy controls (adjusted *p* < 0.001), indicating poorer work ability (Table 2). When WAI categories were compared between groups, healthy controls were more likely to report excellent work ability; whereas individuals with lower limb OA were more likely to report poor or moderate work ability (Table 2; *p* < 0.001).

**Table 2:** Work-related outcomes for participants with lower limb OA and controls

Variable (WAI)	OA group	Control group	*P* value	Adjusted *P* value
WAI score, 7–49	35.0 (30.5, 39.5)	44.5 (41, 47)	< 0.001^a^	< 0.001^b^
Work ability categories, *n* (%)				
Excellent (44–49 points)	5 (4.1)	56 (52.8)	< 0.001^c^	
Good (37–43 points)	45 (36.3)	43 (40.6)		
Moderate (28–36 points)	52 (41.9)	6 (5.7)		
poor (7—27 point)	22 (17.7)	1 (0.9)		
**Variable (WHO-HPQ)**				
Absolute absenteeism, hours/last month	0 (-8, 20)	0 (-12, 14.5)	0.40^a^	0.80^b^
Absolute presenteeism, %	80 (70, 80)	80 (70, 90)	< 0.001^a^	< 0.001^b^
**Variable (WRFQ)**				
WRFQ total score, %	39.8 (27, 81)	50.0 (28, 95)	0.08^a^	0.10^b^
Work scheduling demands, %	43.7 (25, 75)	61.4 (25, 100)	0.05^a^	0.05^b^
Work output demands, %	41.6 (25, 75)	50.0 (25, 96)	0.23^a^	0.10^b^
Physical demands, %	50.0 (30, 70)	65.0 (45, 100)	0.001^a^	0.003^b^
Mental and social demands, %	37.5 (21, 86)	50.0 (25, 96)	0.16^a^	0.30^b^
Flexibility demands, %	40.0 (25, 90)	50.0 (25, 95)	0.19^a^	0.30^b^

All values are presented as median (interquartile range; first and third quartile) unless otherwise stated

^a^Kruskal-Wallis test

^b^Adjusted for age, BMI and physical effort at work using univariate analysis of variance (ANOVA)

^c^Chi-square test

*n* Number, *WAI* Work Ability Index, *WHO-HPQ* World Health Organization’s Health and Work Performanc, *WRF* Work Role Functioning Questionnaire, Participants number for each outcome are: WAI (OA group: 124; control group: 106), WHO-HPQ (OA group: 110; control group: 106), WRFQ (OA group: 101; control group: 98)

There was no statistical difference in absolute absenteeism between OA and control groups (adjusted *p* = 0.80; Table 2). Absolute presenteeism was lower in the OA group than the control group, meaning that individuals with lower limb OA had greater loss of work performance compared with healthy controls (adjusted *p* < 0.001; Table 2).

There was no significant difference in the total WRFQ score between the OA and control groups (adjusted Table 2; *p* = 0.10). When comparing scores in the WRFQ sub-scales, individuals with lower limb OA had more difficulty with work scheduling demands (adjusted *p* = 0.05) and physical demands (adjusted *p* = 0.003) than control participants, but there were no differences in the work output demands, mental and social demands, and flexibility demands between groups (all adjusted *p* ≥ 0.10; Table 2).

## Discussion

This study investigated work-related issues in individuals with and without lower limb OA using a comprehensive suite of outcomes. Individuals with lower limb OA reported poorer work ability (WAI), greater loss of work performance (WHO-HPQ), and more difficulty in performing work scheduling demands and physical demands (WRFQ) compared to similarly aged controls. These differences remained after adjustment for age, BMI, and physical job demands. Absenteeism and the degree of difficulty in performing work output demands, occupational mental and social demands, and flexibility demands were similar between groups. Thus, our data suggests that people with lower limb OA have poorer work participation than people without lower limb OA.

More than half of the workers with lower limb OA in our study had poor or moderate scores for work ability (measured with the WAI); whereas, over half of workers without lower limb OA had excellent work ability. Similarly, a recent study on construction workers with knee OA found poor or moderate work ability in half of participants [38]. There are a number of factors that may contribute to lower work ability in individuals with lower limb OA. First, people with OA in our study had higher physical and mental work demands than those without OA. A positive relationship between physical demands and poorer work ability has been reported in people with knee, hip, hand and spine OA [27]. Second, individuals with lower limb OA have a higher number of comorbidities than controls, as identified in our study sample and reported in previous research [39]. Among workers aged 40 to 65 years, the presence of comorbidities increases the risk of having poor or moderate work ability [40]. Thus, the combination of OA and other comorbidities, along with high physical and mental job demands, may place individuals at greater risk of poor work ability. This is concerning as it has been suggested that workers with poor work ability could be at risk of early retirement from the workforce and future disability pension [41, 42].

Our participants with lower limb OA reported greater loss of work performance (presenteeism) than those without OA. This finding is consistent with previous work that found individuals with OA (anywhere in the body) had greater loss of work performance than controls [43]. A probable explanation for this is that the pain and disability associated with lower limb OA (and OA in other bodily locations) negatively affects performance at work [44]. People may choose to come to work, rather than to take sick leave, but their work performance is not as high as it would be without the physical and mental disability that accompanies lower limb OA [4, 45, 46]. While the WHO-HPQ is a valid measure of presenteeism and widely used to quantify productivity loss, it is a self-report measure that does not capture the actual work performance of an individual [44]. Presenteeism has been suggested to be the primary source of indirect costs to industry, which forms the majority of the economic burden of OA [7].

We did not find any difference in absenteeism (calculated as days absent in one month) between individuals with and without lower limb OA. There is mixed evidence in the literature regarding absenteeism in people with lower limb OA. A cross-sectional study reported similar absenteeism in individuals with early-stage hip and knee OA compared to the general population [47], and a systematic review found low absenteeism in workers with OA [8]. In contrast, people with hip and knee OA awaiting total joint replacement are reported to take more sick leave than a reference population in the year before surgery [14]. Our study population was recruited from the community and were not specifically seeking medical care for their OA, which may explain the difference between study findings. Absenteeism may be affected by severity of OA and further research is needed to investigate this. It is also possible that absenteeism is not different between lower limb OA and control groups because people with OA come to work irrespective of symptoms and disability, but are unable to perform at full capacity. This is consistent with our finding of loss of work performance in individuals with lower limb OA and systematic review findings that presenteeism was four times greater than absenteeism among workers with OA [8]. Finally, the lack of difference in absenteeism could be due to the high physical activity levels in our sample and the lack of difference in physical activity between groups, as physical function has been identified as protective of work loss [16].

People with lower limb OA had more difficulty in meeting work scheduling demands and work physical demands than controls. Work scheduling demands include working without needing to take extra breaks or rest, ease of starting work at the beginning of the day, and sticking to a routine [31]. People with OA report taking extra breaks, arriving late to work and leaving work early [48]. The need to change position regularly and/or decrease load on the affected joint during work to manage pain and symptoms, would be expected to negatively impact work scheduling demands. Work physical demands include sitting, standing, staying in one position for longer than 15 min (reported by 35.5% of participants) and repeating the same motions (e.g., lift, carry, or move objects reported by 15.5% of participants). As static postures and repetitive load activities are aggravating factors for lower limb OA [49], it is not surprising that individuals with lower limb OA have more difficulties performing these work activities. Further, individuals with lower limb OA in our study reported greater physical effort at work than controls. This may be because they had difficulty performing physical tasks, which has previously been reported in workers with musculoskeletal pain [50].

Interestingly, we did not find a difference in total work functioning between workers with and without lower limb OA. This may be because work functioning in our control group was lower than that reported in previous studies [31], possibly due to data collection occurring during COVID-19 pandemic, which is known to negatively affect the mental, physical and work-related health and quality of life [51].

This is one of the first studies specifically designed to understand the different aspects of work participation in people with lower limb OA. Most previous research on lower limb OA has only considered work as secondary outcomes, has not accounted for potential confounding factors, and has focused on hip and knee OA. Our study considers all joints of the lower limb (which have similar impairments and disability) and accounts for confounding factors (e.g., age, BMI, and physical work demands) in analysis. Despite these strengths, there are also limitations to consider. First, OA diagnosis was based on self-report of a diagnosis of lower limb OA by a medical practitioner or the NICE clinical diagnostic guidelines. OA was not confirmed radiographically, and radiographic severity of OA was not considered in analysis. This is a direction for future research. Second, data on work-related variables were self-report and not objectively verified. (e.g., sick leave database). Data obtained about absenteeism was not specific to OA but general physical and mental health. Third, the majority of lower limb OA participants had knee OA (n = 62) with fewer participants having hip, ankle or foot OA (n = 39). Further research is needed to investigate work outcomes in people with hip, ankle and foot OA. Fourth, the study sample size (230 participants) may limit generalization of findings. Many individuals who responded to study advertisement were not eligible for participation, primarily due to the presence of pain elsewhere in the body that was worse than that in the OA joint in individuals with OA, and the presence of bodily pain in the control group. Finally, while our study has identified limitations in work, future research is needed to provide a deeper understanding of why these limitations occur and how people manage them.

Data from our study demonstrates concerns around work ability, lost performance, work scheduling and physical work demands in people with lower limb OA. These factors need to be addressed to facilitate workers with lower limb OA remaining in the workforce. Healthcare professionals and employers are key stakeholders in supporting individuals with OA to remain at work [52, 53]. The NICE guidelines [54] highlight the need for an occupational assessment in the holistic management of a patient with OA. Our findings suggest that an occupational assessment should include talking to patients with lower limb OA about work-related difficulties, specifically in relation work ability, lost performance, work scheduling and physical work demands.

In conclusion, this study identified reduced work ability, lost performance, and difficulties with work scheduling and physical work demands in individuals with lower limb OA compared to controls without OA. Health professionals and employers should consider these challenges when managing patients with lower limb OA and supporting them to remain in the workforce.

## Data Availability

The datasets analysed in the current study are available from the corresponding author on reasonable request.
